# Prevalence of musculoskeletal pain among computer users working from home during the COVID-19 pandemic: a cross-sectional survey

**DOI:** 10.1186/s43161-022-00110-x

**Published:** 2022-12-30

**Authors:** Lakshita Gosain, Irshad Ahmad, Moattar Raza Rizvi, Ankita Sharma, Shobhit Saxena

**Affiliations:** Department of Physiotherapy, Faculty of Allied Health Sciences, Manav Rachna International Institute and Studies (MRIIRS), Faridabad, 121001 India

**Keywords:** COVID-19, Musculoskeletal disorders, Musculoskeletal pain, Worker, Work from home, Nordic Musculoskeletal Questionnaire

## Abstract

**Background:**

Office employees are at a greater risk for musculoskeletal disorders (MSD) due to their prolonged computer use. In the context of COVID-19, an unanticipated shift to working from home is likely to increase MSD due to a lack of an ergonomic workspace and longer workdays.

**Aim:**

To explore the prevalence of MSD with work-related risk factors among the computer users working from home during the COVID-19 pandemic.

**Study design:**

Cross-sectional survey.

**Methods:**

Computer users working from home for more than 6 hours per day during the COVID-19 lockdown were assessed for possible work-related MSD using web-based survey—Google forms for Nordic Musculoskeletal Questionnaire.

**Results:**

One hundred twenty one responses from 53 (43.8%) females (25.47 ± 5.72 years) and 68 (56.2%) males (28.65 ± 4.68 years) were included. The female participants were more prone to musculoskeletal pain as compared to males. The neck pain (60.3%), lower back pain (59.5%), and shoulder pain (49.6%) were the most reported body regions affected by work-related MSD. The elbow (18.2%), wrist/hand (35.5%), upper back (42.1%), hips (24.8%), knee (23.1%), and ankle/feet (14%) were the least affected regions. The risk factors associated with MSP includes lack of workplace at home during lockdown, stress after being in one posture, stress in eyes, and mental stress due to work.

**Conclusions:**

During the lockdown phase, the transition of computer workers from working office environment to home increased the prevalence of MSD more commonly in females than males. There is a need to investigate early detection, prevention, and management options to enhance health outcomes.

## Background

In response to COVID-19 pandemic, the Indian government has instructed people to stay at home and reduce the activities outside the home. As a part of this, many corporates and offices have shifted to working from home, encouraging workers to telecommute wherever possible [1]. Work from home is now a dream come true for the current generation [2]. Keeping this in mind, many office workers have been doing work from home, and in during the sudden lockdown, many professionals lack adequate working environment which further leads to work-related musculoskeletal disorders (which is very common in computer users) which may lead to unintended consequences such as reduced physical activity and faulty postures resulting in worsening of health conditions [3]. According to the National Institute for Occupational Safety and Health, musculoskeletal disorders (MSD) are a damage that affects the musculoskeletal system of the human body, especially at the bones, spinal discs, joints, tendons, ligaments, nerve, cartilages, and blood vessels [4]. MSD include injuries to the soft tissues of the neck, shoulder, elbow, wrist, hand, fingers, back, knee, and ankle [5].

Computer work is widely perceived as a new risk factor for musculoskeletal disorders (MSD) which have become the most frequently diagnosed occupational disease in India. Office workers are at higher risk for musculoskeletal disorders as they spend long time working in front of computers [5]. Soft tissue injuries have increased with the number of people who use computers [6]. Computer work is dangerous because it combines biomechanical factors like static muscle overload [7], repetitive motions, and conditions related to the workplace. Pain and stiffness in the neck, back, shoulders, wrists, and other parts of the body are the most common types of musculoskeletal complaints. People often associate these problems with getting older, but they can happen to both young and old computer users because of things like bad component design, being too close to the screen, and working too many hours in a row. According to several studies, it has been shown that there is a relationship between computer use and MSDs [8]. In the study done by Oha, prevalence rates of musculoskeletal pain for 12 months were found out be 55–69% for the neck, 31–54% for the lower back, and 15–52% for upper extremities. It has been reported that the lower back, neck, and shoulders are the most prone areas in WMSDs [9].

Recent developments in technology and communication have made it possible for more people to work remotely, a trend known as telework. Individuals who telework report a variety of positive outcomes, including reduced stress from reduced commute times, increased productivity from less interruptions, fewer sick days, and, depending on the company, more freedom to prioritize personal and professional obligations. Despite the advantages, it present a number of challenges. Major issues include overworked employees who may come to work sick or disregard physical symptoms and a lack of safety gear [10, 11]. Several psychological issues, including a sense of isolation and potential friction between job and family life, have also been cited [12]. There may have been a decrease in concern for workplace safety and ergonomics [13] due to the unexpected transition to telework for an undetermined period in the context of COVID-19. Because of this, the rapid transition to teleworking may have an effect on the health of the muscles and joints.

Workplace ergonomics is another aspect related to MSP. In the case of cognitive activity with little physical demands, there are a number of ideas concerning the processes of pain etiology, but the precise mechanism is yet unknown [14, 15]. However, many people have discovered a connection between workplace ergonomics and the occurrence of MSD [16]. We expect that working from home will have an impact on work-related MSD since there is some evidence that ergonomic workstations can reduce MSD [17, 18]. This is because most houses lack ergonomic workstations [19].

During the pandemic wave, individuals have adjusted to lengthy hours of computer and laptop usage, such as for work, school, and home entertainment. The high frequency of computer-related MSDs among computer workers indicates a need in public health to develop strategies that lower the intensity of symptoms and minimize disability. Therefore, the aim is to evaluate the work-associated risk factors for musculoskeletal pain in computers users who are working from home during this pandemic.

## Materials and methods

### Study design

A cross-sectional descriptive analytical study was chosen since it was the appropriate design for quickly answering the research questions to assess the prevalence musculoskeletal disorders among computer users working from home. At the same time, it offers the optimum design for answering inquiries during the COVID-19 phase. Snowball sampling technique was used for selection of participants.

### Participants

#### Recruitment and sample size estimation

Sample size was calculated using following formula

$$Sample\ size=\frac{Z_{1-\propto /{2}^2}p\left(1-p\right)}{d^2}$$

where *Z*_1 −  ∝ /2_ = is standard normal variate (at 5 % type 1 error *p* < 0.05, it is 1.96)

*p* = expected proportion in population based on previous studies [20] reporting prevalence of musculoskeletal disorder to be 91% among office workers.

*D* = absolute error or precision of 5% and at type 1 error of 5%.

$${\displaystyle \begin{array}{c} Sample\kern0.17em size=\frac{1.96^2\ast 0.91\left(1-0.09\right)}{0.05^2}\\ {} Sample\kern0.17em size=126\end{array}}$$

**Fig. 1:**
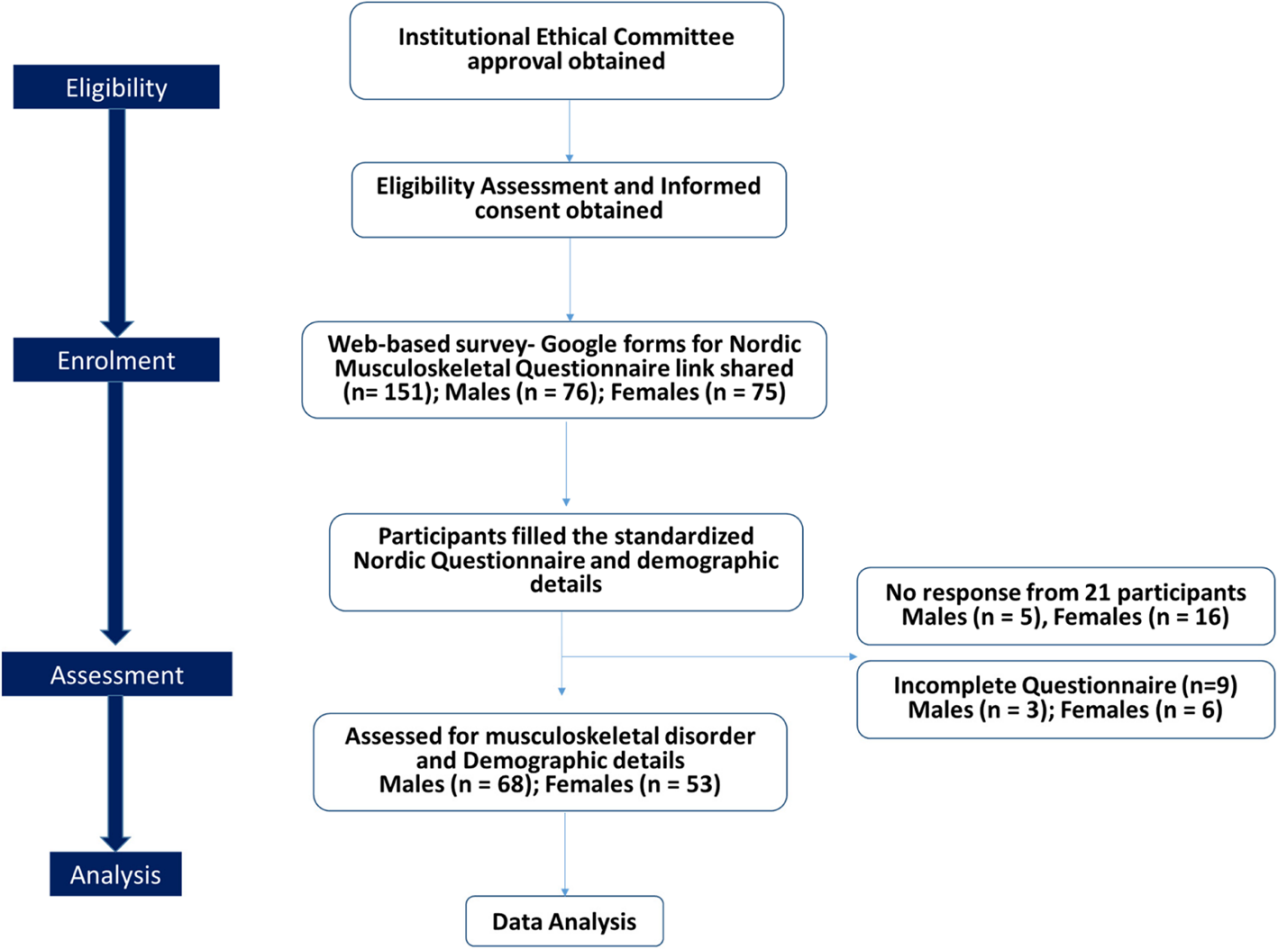
Study flow diagram

Considering 20% drop out, 25 more participants were included making it a total of 151 (Fig. 1).

The questionnaire of nine participants were not considered, as it was not completely filled. The participants were recruited by contacting their organization’s occupational health and safety officers. Four organizations having at least 50 employees working for a period of at least 1 year were chosen for the dissemination of online questionnaire. Participation in this study was anonymous and voluntary.

#### Inclusion criteria

Inclusion criteria were participants working from home using desktop/laptop, above 25 years of age, working for ≥ 6 h and able to understand the English language, and length of employment in current job for at least 12 months. The participation was voluntary and noncommercial.

#### Exclusion criteria

The participants who were working form both office and home, having history of neurological disorders, orthopedic or inflammatory conditions, undergone any recent surgery, and found positive for COVID in last 3 months were excluded from the study.

### Procedure

#### Ethical consideration

This study was conducted at Manav Rachna International Institute of Research and Studies, Department of Physiotherapy, Faculty of Allied Health Sciences. The Departmental Ethical Committee of the Faculty of Allied Health Sciences granted ethical clearance with reference number MRIIRS/FAHS/DEC/2021-BPT016. Each study participant provided written informed permission.

This was an online cross-sectional survey done via “google forms.” Informed consent and questionnaire were attached in one form. The digital link was circulated via social media sites (Facebook and LinkedIn) through which the people of selected organization willing to participate voluntarily responded to the given questionnaire.

### Assessments and outcome measures

#### Demographic characteristics

This includes gender, age, educational qualification, working at which post, and others.

#### Assessment tool

The Nordic Musculoskeletal questionnaire, which has received international recognition and validation, finds symptoms in the neck, back, shoulders, and extremities, was used in this study [21]. The questionnaire is divided into two parts, the general and the specific. The specific part of the questionnaire was modified so that it complies with the circumstances and the context of this research. This included working hours, workplace before lockdown, availability of dedicated workspace, schedules of breaks, working posture, staying physically active, stressed after being in one posture, stress in eyes, mental stress due to work, and healthy work–life balance.

The general part included the Nordic musculoskeletal questionnaire that consists of questions with Yes/No answers about any musculoskeletal symptoms during the last 12 months or the last 7 days and about the impact on activities during the last 12 months. All of these questions referred to nine different bodily regions (the neck, shoulders, elbows, wrists/hands, upper back, lower back, hip/thighs, knees, and ankles/feet) [21, 22].

### Statistical analysis

The data was analyzed using the IBM SPSS 25 version software (IBM Co., Armonk, NY, USA). Shapiro-Wilk test showed that all the data generated from the independent variables were normally distributed (*p* > 0.05). The descriptive statistics, chi-square test, and univariate logistic regression were used to validate the statistical significance between work-associated risk factor and musculoskeletal pain. The statistical significance was indicated if *p* < 0.05 and confidence interval was set at 95%.

## Results

Based on the sample size calculation, the questionnaires were disseminated to around 151 computer professionals working in four organization with minimum employability of 50 staffs; however, only 130 staffs returned the questionnaire. The response rate was 86%. Further, out of 130 responses, nine responses were not considered due to incompleteness, and only 121 responses fulfilled the research criteria.

**Fig. 2:**
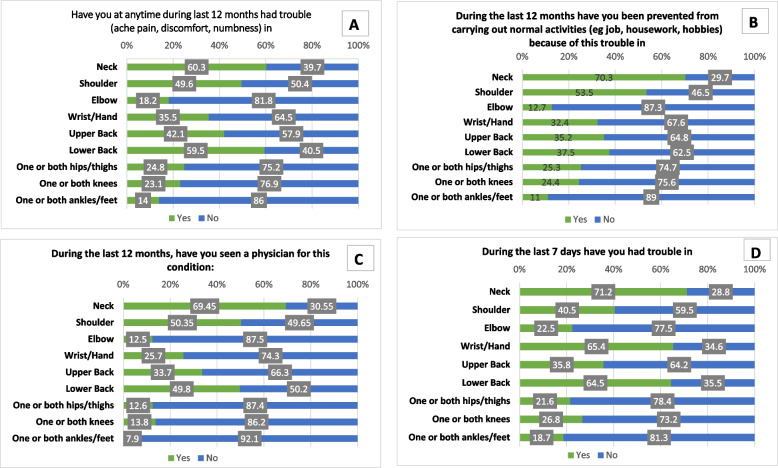
Frequency of musculoskeletal pain reported by the participants

Table 1 shows the frequency distribution of participants work-related details and self-reported MSP. Frequency of musculoskeletal pain based on gender is depicted in Fig. 2. Of all the musculoskeletal pain reported in participants included in the study, pain in the neck (60.3%), shoulder (49.6%), upper back (42.1%), and lower back (59.5%) were found to be more prevalent. However, during last 7 days, the prevalence is higher for the neck (71.2%), wrist/hand (65.4%), lower back (64.5%), and shoulder (40.5%). While answering to the specific musculoskeletal pain preventing the computer workers in their activity, neck (70.3%) and shoulder (53.5%) pain were found to be the main reason. Further, they went to seek medical consultation mainly for neck (69.4%), shoulder (50.35%) and lower back (49.8%).

**Table 1:** Frequency distribution about participants

Characteristics		Frequency (percent)
**Gender**	Female	53 (43.8%)
	Male	68 (56.2%)
**Educational qualification**	Bachelor’s	51 (42.15%)
	Masters	70 (57.85%)
**Workplace before lockdown**	Office	98 (81%)
	Home	23 (19%)
**Working hours from home**	Less than 6 h	11 (9.1%)
	6–8 h	26 (21.5%)
	More than 8 h	84 (69.4%)
**Dedicated workspace**	Yes	61 (50.4%)
	No	60 (49.6%)
**Working posture**	Half lying	32 (26.4%)
	Sitting	87 (71.9%)
	Lying	2 (1.7%)
**Regular breaks**	Yes	78 (64.5%)
	No	43 (35.5%)
**Schedules of breaks**	0 break	3 (2.5%)
	1 break	42 (34.7%)
	2-5 breaks	61 (50.4%)
	>5 breaks	15 (12.4%)
**Stay physically active**	Yes	65 (53.7%)
	No	56 (46.3%)
**Stressed after being in one posture**	Yes	58 (47.9%)
	No	12 (9.9%)
	Sometimes	51 (42.1%)
**Stressed in eyes**	Yes	80 (66.1%)
	No	10 (8.3%)
	Sometimes	31 (25.6%)
**Mental stress due to work**	Yes	48 (39.7%)
	No	73 (60.3%)
**Healthy work life balance**	Yes	61 (50.4%)
	No	60 (49.6%)

### Prevalence of musculoskeletal pain (MSP) in respect to work-associated risk factor

Prevalence of MSP in respect to work-associated risk factor is illustrated in Tables 2, 3, and 4. Chi-square test showed that there was a significant difference in the prevalence of neck pain between genders (*p* = 0.03). Regarding work details, there was a significant difference in the prevalence of neck pain between being stressed when stick to one posture (*p* = 0.05), between stress in eyes due to increased screen time (*p* = 0.03), and between mental stress due to work from home (*p*= 0.002). There was a significant difference in the prevalence of upper back pain between genders (*p* <0.01). Regarding work details, there was a significant difference in the prevalence of upper back pain between workplace before lockdown (*p* = 0.035) and between mental stress due to work from home (*p* = <0.01). In addition, there was a significant difference in the prevalence of low back pain (LBP) between workplace before lockdown (*p* = 0.010), between staying physically active (*p* = 0.042), between stress in eyes due to increased screen time (*p* = 0.029), and between mental stress due to work from home (*p* = 0.002) (Table 2).

**Table 2:** Prevalence of neck and back musculoskeletal pain in respect to work-associated risk factor

Variables	Neck			Upper back			Lower back		
	Present ***n*** (%)	Absent ***n*** (%)	χ2 (***p*** value)	Present ***n*** (%)	Absent ***n*** (%)	χ2 (***p***-value)	Present ***n*** (%)	Absent ***n*** (%)	χ2 (***p***-value)
**Gender**									
Female	40 (33.0)	13 (10.7)	9.03 (.003*)	35 (28.9)	18 (14.9)	22.07 (< .001*)	37 (30.6)	16 (13.2)	4.15 (.061)
Male	33 (27.3)	35 (28.9)		16 (13.2)	5 2 (43.0)		35 (28.9)	33 (27.3)	
**Workplace before lockdown**									
Office	60 (49.6)	38 (31.4)	.172 (.813)	46 (38.0)	52 (43.0)	4.851 (.035*)	64 (52.9)	34 (28.1)	7.202 (.010*)
Home	13 (10.7)	10 (8.3)		5 (4.1)	18 (14.9)		8 (6.61)	15 (12.4)	
**Working hours from home**									
< 6 h	7 (5.8)	4 (3.3)	1.267 (.531)	3 (2.5)	8 (6.6)	2.55 (.279)	5 (4.1)	6 (4.5)	2.673 (.263)
6–8 h	18 (14.9)	8 (6.6)		14 (11.6)	12 (9.9)		13 (10.7)	13 (10.7)	
> 8 h	48 (39.7)	36 (29.7)		34 (28.1)	50 (41.3)		54 (44.6)	30 (24.8)	
**Dedicated workspace**									
Yes	29 (24.0)	32 (26.4)	3.185 (.095)	23 (19.0)	38 (31.4)	.996 (.360)	27 (22.3)	34 (28.1)	.724 (.460)
No	41 (33.9)	19 (15.7)		32 (26.4)	28 (23.1)		38 (31.4)	22 (18.2)	
**Working posture**									
Half lying	22 (18.2)	10 (8.3)	2.84 (.241)	16 (13.2)	16 (13.2)	2.39 (.302)	21 (17.4)	11 (9.1)	.722 (.697)
Sitting	49 (40.5)	38 (31.4)		35 (28.9)	52 (43.0)		50 (41.3)	37 (30.6)	
Lying	2 (1.7)	0 (0.00)		0 (0.00)	2 (1.7)		1 (0.8)	1 (0.8)	
**Regular breaks**									
Yes	26 (21.5)	17 (14.0)	.001 (1.000)	20 (16.5)	23 (19.0)	.521 (.565)	26 (21.5)	17 (14.0)	.026 (1.000)
No	47 (38.8)	31 (25.6)		47 (38.8)	31 (25.6)		46 (38.0)	32 (26.4)	
**Schedules of break**									
0 break	0 (00.0)	3 (2.5)	3.489 (.322)	3 (2.5)	0 (0.00)	5.79 (.122)	2 (1.7)	1 (0.8)	1.74 (.627)
1 break	15 (12.4)	27 (22.3)		19 (15.7)	23 (19.0)		19 (15.7)	23 (19.0)	
2–5 break	28 (23.1)	33 (26.4)		25 (21.0)	36 (29.7)		22 (18.2)	39 (32.2)	
> 5 breaks	5 (4.1)	10 (8.3)		4 (3.3)	11 (9.1)		6 (4.5)	9 (7.4)	
**Stay physically active**									
Yes	35 (28.9)	30 (24.8)	2.468 (.138)	25 (21.0)	40 (33.0)	.783 (.461)	33 (27.3)	17 (14.0)	4.44 (.042*)
No	38 (31.4)	18 (14.9)		26 (21.5)	30 (24.8)		39 (32.2)	32 (26.4)	
**Stressed after being in one posture?**									
Yes	42 (34.7)	16 (13.2)	10.42 (.005*)	29 (24.0)	29 (24.0)	4.84 (.089)	38 (31.4)	20 (16.5)	4.29 (.117)
No	3 (2.5)	9 (7.9)		2 (1.7)	10 (8.3)		4 (3.3)	8 (6.6)	
Sometimes	28 (23.1)	23 (19.0)		20 (16.5)	31 (25.6)		30 (24.8)	21 (17.4)	
**Stress in eyes**									
Yes	57 (47.1)	23 (19.0)	11.76 (.003*)	39 (32.2)	41 (33.9)	4.685 (.096)	50 (41.3)	30 (24.8)	7.09 (.029*)
No	4 (3.3)	6 (4.5)		2 (1.7)	8 (6.6)		2 (1.65)	8 (6.6)	
Sometimes	12 (9.9)	19 (15.7)		10 (8.3)	21 (17.4)		20 (16.5)	11 (9.1)	
**Mental stress due to work.**									
Yes	37 (30.6)	11 (9.1)	9.33 (.002*)	30 (24.8)	18 (14.9)	13.51 (< .001*)	37 (30.6)	11 (9.1)	10.20 (.002*)
No	36 (29.7)	37 (30.6)		21 (17.4)	52 (43.0)		35 (28.9)	38 (31.4)	
**Healthy work life balance?**									
Yes	36 (29.7)	25 (21.0)	.089 (.853)	27 (22.3)	34 (28.1)	.225 (.714)	35 (28.9)	26 (21.5)	.231 (.712)
No	37 (30.6)	23 (19)		24 (19.8)	36 (29.7)		37 (30.6)	23 (19)	

*Significant difference

**Table 3:** Prevalence of upper limb musculoskeletal pain in respect to work-associated risk factor

Variables	Shoulder			Elbow			Wrist/hand		
	Present ***n*** (%)	Absent ***n*** (%)	χ2 (***p***-value)	Present ***n*** (%)	Absent ***n*** (%)	χ2 (***p***-value)	Present ***n*** (%)	Absent ***n*** (%)	χ2 (***p***-value)
**Gender**									
Female	35 (28.9)	18 (14.9)	10.21 (.002*)	15 (12.4)	38 (31.4)	6.49 (.016*)	29 (24.0)	24 (19.4)	15.144 (< .001*)
Male	25 (21.0)	43 (35.5)		7 (5.8)	61 (50.4)		14 (11.6)	54 (44.6)	
**Workplace before lockdown**									
Office	52 (43.0)	46 (38.0)	2.49 (.164)	16 (13.2)	82 (67.7)	1.19 (.366)	58 (47.9)	40 (33.0)	6.27 (.015*)
Home	8 (6.6)	15 (12.4)		6 (4.5)	17 (14.0)		3 (2.5)	20 (16.5)	
**Working hours from home**									
< 6 h	4 (3.3)	42 (34.7)	8.964 (.018*)	2 (1.7)	9 (7.4)	.179 (.914)	6 (4.5)	55 (45.4)	2.031 (.362)
6–8 h	14 (11.6)	12 (9.9)		4 (3.3)	22 (18.2)		8 (6.6)	5 (4.1)	
> 8 h	42 (34.7)	7 (5.8)		16 (13.2)	68 (56.2)		29 (24.0)	18 (14.9)	
**Dedicated workspace**									
Yes	27 (22.3)	34 (28.1)	1.39 (.277)	10 (8.3)	51 (42.5)	.264 (.644)	18 (14.9)	43 (35.5)	11.952 (.007*)
No	33 (27.3)	27 (22.3)		12 (9.9)	48 (39.7)		35 (28.9)	25 (21.0)	
**Working posture**									
Half lying	15 (12.4)	17( 14.0)	2.12 (.345)	5 (4.1)	27 (22.3)	1.50 (.471)	19 (15.7)	13 (10.7)	1.50 (.471)
Sitting	43 (35.5)	44 (36.3)		16 (13.2)	71 (58.6)		30 (24.8)	57 (47.1)	
Lying	2 (1.7)	0 (00.0)		1 (0.8)	1 (0.8)		0	2 (1.7)	
**Regular breaks**									
Yes	23 (19.0)	20 (16.5)	.406 (.572)	9 (7.4)	34 (28.1)	.339 (.625)	17 (14.0)	26 (21.5)	.465 (.554)
No	41 (33.9)	37 (30.6)		13 (10.7)	65 (53.7)		52 (43.0)	26 (21.5)	
**Schedules of break**									
0 break	2 (1.7)	1 (0.8)	2.57 (.462)	0 (0.00)	3 (2.5)	2.39 (.495)	0 (0.00)	3 (2.5)	4.478 (.214)
1 break	24 (19.8)	18 (14.9)		9 (7.4)	33 (27.3)		19 (15.7)	23 (19)	
2–5 break	26 (21.5)	35 (28.9)		12 (9.9)	49 (40.5)		18 (14.9)	43 (35.5)	
> 5 breaks	8 (6.6)	7 (5.8)		1 (0.8)	14 (11.6)		6 (4.5)	9 (7.4)	
**Stay physically active**									
Yes	2 5 (21.0)	31 (25.6)	1.38 (.276)	10 (8.3)	55 (45.4)	.739 (.480)	22 (18.2)	43 (35.5)	.175 (.707)
No	36 (29.7)	29 (24.0)		12 (9.9)	44 (36.3)		21 (17.4)	35 (28.9)	
**Stressed after being in one posture?**									
Yes	37 (30.6)	21 (17.4)	13.70 (.001*)	12 (9.9)	46 (38.0)	2.98 (.225)	28 (23.1)	30 (24.8)	7.900 (.019*)
No	1 (0.8)	11 (9.1)		0 (0.00)	12 (9.9)		3 (2.5)	9 (7.4)	
Sometimes	22 (18.2)	29 (24.0)		10 (8.3)	41 (33.9)		12 (9.9)	39 (32.2)	
**Stress in eyes**									
Yes	52 (43.0)	28 (23.1)	22.43 (< .001*)		60 (49.6)	7.611 (0.22)	36 (29.7)	44 (36.3)	9.278 (.010*)
No	2 (1.7)	8 (6.6)		1 (0.8)	9 (7.4)		2 (1.7)	8 (6.6)	
Sometimes	6 (4.5)	25 (21.0)		1 (0.8)	30 (24.8)		5 (4.1)	26 (21.5)	
**Mental stress due to work**									
Yes	34 (28.1)	14 (11.6)	14.36 (< .001*)	11 (9.1)	37 (30.6)	1.19 (.337)	28 (23.1)	20 (16.5)	18.04 (< .001*)
No	26 (21.5)	47 (38.8)		11 (9.1)	62 (52.0)		15 (12.4)	58 (47.9)	
**Healthy work life balance?**									
Yes	26 (21.5)	35 (28.9)	2.386 (.147)	7 (5.8)	54 (44.6)	3.719 (.062)	18 (14.9)	43 (35.5)	1.952 (.187)
No	34 (28.1)	26 (21.5)		15 (12.4)	45 (37.2)		25 (21.0)	35 (28.9)	

*Significant difference

**Table 4:** Prevalence of lower limb musculoskeletal pain in respect to work-associated risk factor

Variables	Hips/thighs			Knee			Ankle/feet		
	Present ***n*** (%)	Absent ***n*** (%)	χ2 (***p***-value)	Present ***n*** (%)	Absent ***n*** (%)	χ2 (***p***-value)	Present ***n*** (%)	Absent ***n*** (%)	χ2 (***p***-value)
**Gender**									
Female	14 (11.6)	39 (32.2)	.133 (.832)	21 (17.4)	32 (26.4)	14.40 (< .001*)	12 (9.9)	41 (33.9)	5.76 (.019*)
Male	16 (13.2)	52 (43.0)		7 (5.8)	61 (50.4)		5 (4.1)	63 (52.0)	
**Workplace before lockdown**									
Office	26 (21.5)	72 (59.5)	.834 (.432)	26 (21.5)	72 (59.5)	3.32 (.098)	13 (10.7)	85 (70.0)	.263 (.738)
Home	4 (3.3)	19 (15.7)		2 (1.7)	21 (17.4)		4 (3.3)	19 (15.7)	
**Working hours from home**									
< 6 h	2 (1.7)	65 (53.7)	1.81 (.403)	3 (2.5)	63 (52.0)	1.148 (.563)	1 (0.8)	71 (58.6)	.501 (.778)
6–8 h	9 (7.4)	17 (14.0)		4 (3.3)	22 (18.2)		3 (2.5)	23 (19)	
>8 h	19 (15.7)	9 (7.4)		21 (17.4)	8 (6.6)		13 (10.7)	10 (8.3)	
**Dedicated workspace**									
Yes	12 (9.9)	49 (40.5)	13.73 (.002*)	14 (11.6)	47 (38.8)	14.02 (< .001*)	9 (7.4)	52 (43.0)	0.51 (1.000)
No	42 (34.7)	18 (14.9)		46 (38.0)	14 (11.6)		52 (43.0)	8 (6.6)	
**Working posture**									
Half lying	10 (8.3)	22 (18.2)	1.527 (.466)	8 (6.6)	24 (19.8)	.665 (.717)	6 (4.5)	26 (21.5)	1.05 (.590)
Sitting	20 (16.5)	67 (55.3)		20 (16.5)	67 (55.3)		11 (9.1)	76 (62.8)	
Lying	0 (0.00)	2 (1.7)		0 (0.00)	2 (1.7)		0 (0.00)	2 (1.7)	
**Regular breaks**									
Yes	14 (11.6)	64 (52.9)	5.51 (.027*)	17 (14.0)	61 (50.4)	.223 (.657)	9 (7.4)	69 (57.0)	1.14 (.290)
No	27 (22.3)	16 (13.2)		32 (26.4)	11 (9.1)		35 (29.9)	8 (6.6)	
**Schedules of break**									
0 break	1 (0.8)	2 (1.7)	.301 (.960)	0 (0.00)	3 (2.5)	2.41 (.491)	1 (0.8)	2 (1.7)	1.11 (.773)
1 break	11 (9.1)	31 (25.6)		12 (9.9)	30 (24.8)		5 (4.1)	37 (30.6)	
2–5 break	14 (11.6)	47 (38.8)		14 (11.6)	47 (38.8)		9 (7.4)	52 (43.0)	
>5 breaks	4 (3.3)	11 (9.1)		2 (1.7)	13 (10.7)		2 (1.65)	13 (10.7)	
**Stay physically active**									
Yes	17 (14.0)	48 (39.7)	.139 (.833)	12 (9.91)	53 (43.8)	1.729 (.203)	8 (6.6)	57 (47.1)	.353 (.607)
No	43 (35.5)	13 (10.7)		40 (33.0)	16 (13.2)		47 (38.8)	9 (7.4)	
**Stressed after being in one posture?**									
Yes	18 (14.9)	40 (33.0)	3.24 (.198)	15 (12.4)	43 (35.5)	.595 (.743)	12 (9.9)	46 (38.0)	4.841 (.089)
No	1 (0.8)	11 (9.1)		2 (1.65)	10 (8.3)		0 (0.00)	12 (9.9)	
Sometimes	11 (9.1)	40 (33.0)		11 (9.1)	40 (33.0)		5 (4.1)	46 (38.0)	
**Stress in eyes**									
Yes	22 (18.2)	58 (47.9)	1.56 (.456)	24 (19.8)	56 (46.3)	6.95 (.031*)	1 7 (14.0)	63 (52.0)	10.13 (.006)
No	1 (0.8)	9 (7.4)		0 (0.00)	10 (8.3)		0 (0.00)	10 (8.3)	
Sometimes	7 (5.8)	24 (19.8)		4 (3.3)	27 (22.3)		0 (0.00)	31 (25.6)	
**Mental stress due to work?**									
Yes	15 (12.4)	33 (27.3)	1.779 (.202)	16 (13.2)	32 (26.4)	4.64 (.046*)	9 (7.4)	39 (32.2)	1.456 (.287)
No	15 (12.4)	58 (47.9)		12 (9.9)	61 (50,4)		8 (6.6)	65 (53.7)	
**Healthy work life balance?**									
Yes	12 (9.9)	49 (40.5)	1.73 (.212)	9 (7.4)	52 (43.0)	4.86 (.032*)	6 (4.5)	55 (45.4)	1.809 (.201)
No	18 (14.9)	42 (34.7)		19 (15.7)	41 (33.9)		11 (9.1)	49 (40.5)	

*Significant difference

There was a significant difference in the prevalence of shoulder pain between genders (*p* = 0.002), between being stressed when stick to one posture (*p* = 0.001), between stress in eyes due to increased screen time (*p* = <0.01), and between mental stress due to work from home (*p* = <0.01). There was a significant difference in the prevalence of elbow pain between genders (*p* = 0.016). Further, there was a significant difference in the prevalence of wrist/hand pain between genders (*p* = <0.01), between workplace before lockdown (*p =* 0.015), between being stressed when stick to one posture (*p* = 0.019), between stress in eyes due to increased screen time (*p* = 0.010), and between mental stress due to work from home (*p* = <0.01) (Table 3).

There was a significant difference in the prevalence of hip/thigh pain between genders (*p* = <0.01) and between regular breaks (*p* = 0.027). Regarding work details, there was a significant difference in prevalence of knee between stress in eyes due to increased screen time (*p*= 0.031), between mental stress due to work from home (*p*= < 0.01), and between healthy work life balance (*p* = 0.032) There was a significant difference in the prevalence of ankle/feet pain between genders (*p* = 0.019) (Table 4).

### Work factors associated with musculoskeletal pain (MSP)

Univariate logistic regression analysis between neck pain and work-related factors showed that neck pain had significant association with those who feel stressed sometimes after being in one posture (*p* = 0.015) and those who feel stressed every time they sit in one posture (*p* = 0.019). Neck pain was also significantly associated with those participants either having stress in eyes (*p* = 0.042) or not having stress in eyes (*p* = 0.012). Univariate logistic regression analysis between upper back pain and work-related factors revealed a strong association of both male and female with upper back pain (*p* = < 0.001). It also showed a significant association of those who feel mental stress due to work (*p* = 0.021) with upper back pain. Further, univariate logistic regression analysis between lower back pain and work-related factors showed that participants had significant association of back pain with workplace either at office or home before lockdown (*p* = 0.004). Again, upper back pain was significantly associated with those who stay physically active (*p* = 0.045), who sometimes feel stress in their eyes (*p* = 0.032) and feel mental stress due to work (*p* = 0.019) with lower back pain.

Univariate logistic regression analysis between shoulder pain and work-related factors showed a significant association of shoulder pain with workplace either officer or home before lockdown (*p* = 0.034) and mental stress due to work (*p* =0.012). Participants who were feeling stress in eyes and those who were not feeling stress in eyes showed a strong association with shoulder pain (*p* = < 0.001). Univariate logistic regression analysis between pain in elbow and work-related factors showed a significant association of pain in elbow with those who were not feeling stress in eyes (*p* = 0.024). Univariate logistic regression analysis between pain in wrists/hands and work-related factors showed a significant association of pain in wrists/hand with gender (*p* = 0.006). Significant association of pain in wrists/hands was also observed with workplace before lockdown (*p* = 0.016), with working hours for less than 6 h (*p* = 0.019) and 6–8 h (*p* = 0.005), with mental stress due to work (*p* = 0.002), and with those who were feeling stress in eyes (*p* = 0.038) and not feeling stress in eyes (*p* = 0.011).

Univariate logistic regression analysis between pain in hip and thighs and work-related factors showed a significant association of pain in hip and thighs with those participants working for more than 8 h (*p* = 0.022) and having no regular breaks (*p* = 0.007). Univariate logistic regression analysis between knee pain and work-related factors showed a significant association of knee pain with gender (*p* = 0.009). Similarly, univariate logistic regression analysis between ankle/feet pain and work-related factors showed a significant association of ankle/feet pain with gender (*p* = 0.045).

## Discussion

Research on the effects of the COVID-19 pandemic necessitates a wide range of data. Even without the addition of COVID-specific questions, repeat polls, retrospective surveys, and demographic registries are valuable for observing pauses in trends throughout the crisis and variances among nations. The present study was aimed to investigate the prevalence of MSD and work-associated risk factor in computer user a working from home. The Standardized Nordic Questionnaire [21] was used to evaluate the musculoskeletal pain in computer users. Work-related details were also asked to evaluate the risk factors responsible for MSD. Given that MSD is a biopsychosocial phenomenon [23, 24] and that prior research indicates that the home-office people may experience an increase in mental health difficulties and spine pain [25], we anticipated an increased prevalence of MSD during the lockdown. The results of our study shows that MSD is most common in females. As per the results of the present study neck pain, low back pain and shoulder pain were highly affected region in this population. Similar findings has been reported in previous study where the MSP is influenced by gender and more prevalent in females [26]. In another study, it is reported that men and women who work with computers in the office have a 39% and 44% prevalence of neck pain and a 42% and 45% prevalence of lower back pain respectively [4].

In our study, it was found out that prevalence of neck pain is seen in female, those who stay in one posture for long time and in front of screen for long time that cause stress in eyes and those who are suffering from mental stress while working from home during this pandemic. Prevalence of upper back is also observed more in females working from office before lockdown, now working from home, and suffering from mental stress while working from home in this pandemic. Similar findings regarding musculoskeletal discomfort were observed among female office workstation users who worked from home during the COVID-19 pandemic and had a higher prevalence of physical and mental health problems [27].

The findings of one study from Turkey [28], conducted during a lockdown, found a substantial worsening of lower back pain in persons who worked from home compared to those who continued to work at their regular jobs. However, unlike the Turkish study participants who worked from home (teachers, academics, and students), our participants working on computer peripherals whose job demands frequent computer use demonstrate the higher prevalence of lower back pain worsening Another study in Italy [25] found that 50% of subjects experienced worsening neck pain. Females with wrist/hand pain were more likely to be working from home before the lockdown, stressed from being seated, stressed from staring at a screen, and stressed from work and the pandemic. Participants who do not take regular breaks have more hip/thigh pain. Females with eyestrain from constant screen staring, mental stress from work, and lack of work–life balance are more prone to knee pain. Female also had ankle pain. Similarly, above parameters are linked to MSD of all body regions.

In the study done by Okezue [29], findings were that women were more affected than males in WMSDs, and 58.1% women were affected by WMSDs which support the finding of our study. Awkward posture, sustained body position, and improper bending had positive relationship with WMSDs. In study done by Kumar [30], it has been found that sustained body position had a significant effect on WMSDs, which put the muscle under stress and lead to fatigue and pain. Participants who worked for more than 7 h exhibited a strong connection with WMSDs. These findings are in line with prior research findings that MSD is most common in females, people who sit for lengthy periods of time, those who worked from home before the lockdown, and those who suffer from work-related stress. The clinical importance of this study is that we can construct ergonomic exercises based on the study’s findings.

As far as various recommendations are concerned, regular breaks from computer work are highly suggested [1, 31], as doing exercises alleviate strain on the spine, neck, upper extremities, and eyes [13]. Due to the abrupt transition to daily work from home, the declines in workplace comfort and ergonomics scores were expected. One possible explanation for this decline is the transition from a desktop computer to a less comfortable laptop computer. Additionally, a correlation has been established between laptop use and a worsening of neck pain during the lockdown [25]. This suggests that enhancing workplace comfort and ergonomics may help avoid MSD.

Given that this new work environment introduces new health and safety risks, particularly those associated with the development or worsening of musculoskeletal pain, we believe that future safety guidelines and precautions are necessary, as is a multifaceted and integrated approach to this issue aimed at improving worker health and minimizing work-related pain [17]. We have come up with a few suggestions in this regard. The solution to the situation from employer can only be by providing an evaluation of the new workplace and ergonomic desk and chair, as well as train employees how to improve their “imperfect” working conditions to make them ergonomically acceptable.

### Strength and limitations of the study

The strength of this study is that if MSD and risk factors are identified at an early stage, it is possible to reduce their incidence and try to prevent them by implementing an ergonomic workstation and suitable work routine. Similar to earlier research, this one had few limitations as well. One of the major drawbacks of this study is that it was conducted during the “lockdown phase of COVID 19.” A comparison would have been extremely helpful had the study been undertaken again after the lockdown. The sample size in the study was small. Participants reported their own responses, which may have affected the results due to misinterpretations of the questions. Because it was conducted online, the survey took a considerable amount of time. There was no way to evaluate the participants in an unbiased manner.

### Future scope of this study

A similar study can be conducted on specific age group to find out the association between age and occurrence of MSD. MSP can be evaluated before and after implementing ergonomic exercise and imparting ergonomic education in their routine. This study can be used further for designing appropriate interventions to prevent MSD.

### Clinical implications of the study

As work from home for computer workers is going to be a new normal in India, the findings of this study can help them to know the major areas of the body that are prone towards MSPs and the importance of work breaks, position of computer use, and working hours to prevent the occurrence of MSD.

## Conclusions

This study shows the incidence of MSP and work-related risk factors among home computer users. The most commonly reported physical pains were neck, lower back, and shoulder. MSP was highly predisposed in female participants who were stressed from being in one position, working from home prior to the pandemic, had extra time, and had elevated mental stress from work. Effective computer user interventions should be designed.

## Abbreviations

MSP: Musculoskeletal pain
MSD: Musculoskeletal disorders
WMSDs: Work-related muscular disorders
*χ*^2^: Chi square
